# Inverted irrigation hook for descemetorhexis in endothelial keratoplasty: Instrument design and surgical technique

**DOI:** 10.5935/0004-2749.2024-0204

**Published:** 2024-08-30

**Authors:** Luiz Felipe Lynch de Moraes, Maria Isabel Lynch Gaete, Marília Rocha Costa, Rodrigo Pessoa Cavalcanti Lira

**Affiliations:** 1 Universidade Federal de Pernambuco, Recife, PE, Brazil; 2 Universidade Federal de São Paulo, São Paulo, SP, Brazil; 3 Universidade Estadual de Campinas, Campinas, SP, Brazil

Dear Editor,

Following the inception of endothelial transplants, especially Descemet membrane endothelial keratoplasty (DMEK), surgical methodologies have significantly evolved. The descemetorhexis technique, has become indispensable in both Descemet’s stripping automated endothelial keratoplasty (DSAEK) and DMEK, facilitating a smoother integration of the graft into the recipient’s stromal interface. Initially, anterior chamber air injections were used for descemetorhexis^(1)^. Subsequently, cohesive viscoelastics^(2)^, anterior chamber maintainers^(3)^, or a combinations of these techniques have been used. Each of these modalities have their distinct advantages and challenges. Given the lack of consensus on a superior technique, we have delineated a straightforward and readily accessible instrument to refine the descemetorhexis technique.

**Figure 1.**
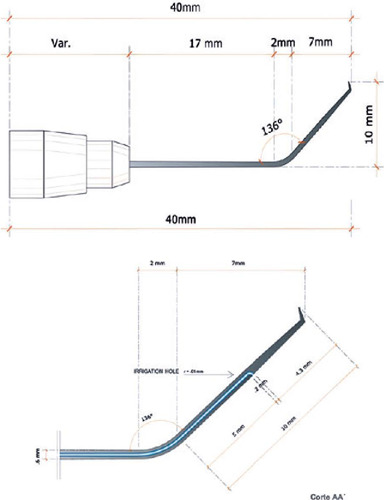
Technical scheme and measurements of the inverted irrigation hook (lateral view).

**Figure 2.**
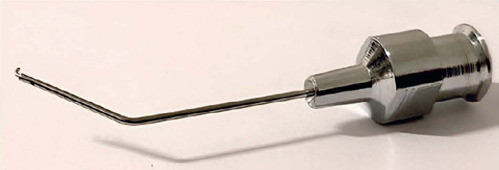
Inverted metal irrigation hook. The posterior orifce allows for the maintenance of the anterior chamber. The orifce was placed closer to the hook than in the hook depicted in fgure 1. The closer the orifce to the tip, the better.

The described instrument is an inverted irrigation hook that has been crafted in a cannula configuration. It obviates the need for viscoelastic application and its subsequent removal or the employment of an anterior chamber maintainer. Technical illustrations, dimensions, and design of the instrument are provided in figure 1. The instrument is available in both disposable and reusable metal forms (Figure 2). A 136° angulation ensures ergonomic operation, mirroring the familiar inverted Sinskey hook and thereby reducing the learning curve associated with its adoption. Its 10-mm length adequately spans the requisite corneal area, and the cannula’s design simplifies its integration with universally compatible serum infusion systems that utilize gravity-fed pressure regulation. The strategic placement of an inferior orifice preserves anterior chamber integrity without supplementary devices, which is essential for maintaining proximity to the hook tip and preventing exterior displacement during incisional procedures and anterior chamber instability.

The irrigated inverted hook is used during desceme-torhexis. Routine practice involves a partial incision, measuring either 2.4 mm or 2.75 mm, and an internal corneal entry point of approximately 1.2-1.5 mm. This will minimize balanced saline solution (BSS) loss and maintain anterior chamber stability. Although surgeon preference dictates paracentesis execution, it is recommended after descemetorhexis. After placing a myostatic, the irrigated inverted hook is coupled to a universal equipment connected to a BSS. The bottle is placed at the height of a standard support, and it can be raised or lowered according to intraoperative needs. Careful insertion and operation of the hook are paramount, especially near the incision, to prevent orifice occlusion and subsequent anterior chamber destabilization. Subsequently, the incision is enlarged and the corneal transplant is inserted.

Variations in the techniques of descemetorhexis exist because it is a new and constantly evolving surgery. Although the use of air simplifies the visualization of the Descemet membrane detachment, it risks anterior chamber lowering and iris or lens contact with the inverted hook. Anterior chamber maintainers offer stability. However, they necessitate additional handling and surgical steps. Viscoelastic agents are the safest choice owing to their ability to maintain anterior chamber stability. Therefore, they are advantageous for intricate surgical scenarios or when treating phakic individuals. Nonetheless, viscoelastic substances are a costlier and more tedious option due to the potential for endothelial graft displacement in instances of incomplete removal^(4,5)^. The inverted irrigated hook mitigates these issues by ensuring anterior chamber stability, reducing ocular manipulation, and decreasing overall costs. For surgeons utilizing viscoelastic agents, it also lessens the risk of graft dislocation due to residual material. In conclusion, herein, we have introduced a novel, cannula-shaped inverted irrigated hook that has been designed for simplicity, affordability, and efficacy in DSAEK/DMEK procedures, with the ultimate aim of reducing costs and complication risks.
